# The Correlation Between Quality of Life and Acceptability of Disability in Patients With Facial Burn Scars

**DOI:** 10.3389/fbioe.2019.00329

**Published:** 2019-11-14

**Authors:** Xiuni Zhang, Yuan Liu, Xiaohong Deng, Chengsong Deng, Yunfeng Pan, Ailing Hu

**Affiliations:** ^1^Department of Trauma and Orthopaedics, Guangzhou Panyu Central Hospital, Guangzhou, China; ^2^Third Affiliated Hospital of Sun Yat-sen University, Guangzhou, China

**Keywords:** quality of life, acceptability of disability, facial, burn scars, nursing

## Abstract

The purpose of our research is to understand the status of the quality of life and level of disability acceptance in patients with facial burn scars and to explore the correlation between quality of life and disability acceptance and how to improve nursing care for these patients. Patients with facial burn scars were investigated in an outpatient clinic of tertiary hospitals from September 2015 to February 2016. A cross-sectional survey was conducted. The questionnaires used included demographic data and investigations using the burn scars table, Burn-Specific Health Scale-Brief (BSHS-B), and acceptance disability scale (ADS). Differences between participants in terms of demographic characteristics, quality of life, and disability acceptance were assessed using two-tailed independent *t*-tests. The total score of quality of life and disability acceptance in facial burn scar patients was 137.06 ± 17.05 and 185.68 ± 23.74, respectively. The results of Spearman correlation analysis showed that the overall quality of life score of facial burn scar patients was positively correlated with disability acceptance (*r* = 0.245, *p* = 0.007). The quality of life of facial burn scar patients will improve with the improvement of disability acceptance level. Therefore, medical staff can improve the quality of life of patients by improving their disability acceptance level.

## Introduction

Burns are generally caused by high-intensity currents, high temperatures, chemicals, physical rays, etc. (Simons et al., 2018; Van Lieshout et al., 2018). With continuous mechanization and urbanization, the incidence of burns continues to increase. Although the government's efforts in prevention and treatment have reduced the mortality of burn, the disability rate of burn patients has not decreased. It is reported that the annual incidence of burns in China is ~2% (Brewin and Homer, 2018), which occupies the second highest mortality rate among accidents. As an obvious exposed part of the body, facial burns account for more than half of all burn incidents.

The loose subcutaneous adipose tissue and complex vascular nerves in facial areas makes it easier for body fluids to accumulate in the interstitial space. At the same time, the body's own oral and nasal secretions increase the incidence of infection in facial burns, resulting in hypertrophic scars or keloids during the tissue repair process (Kowal-Vern and Criswell, 2005). Deep second degree burns usually leave scars of different sizes, and when the wounds are not treated properly, shallow second degree burns or even degree I burns may form scars.

Scars after burns can cause dysfunction and disfigurement, which greatly affects the patient's daily life and social interaction. Patients often feel disappointment, fear, inferiority, anxiety, loneliness, suspiciousness, and mental disorders due to changes in their appearance. Disfigurement can also lead to social escape. Some patients still cannot accept themselves after long-term recovery and even have a suicidal tendency (Yurdalan et al., 2018).

Burns can produce negative emotions such as anxiety and depression, which in turn affect the quality of life (Kowal-Vern and Criswell, 2005; Miller et al., 2013; Cakir et al., 2015; Spronk et al., 2018a). Studies have shown that the quality of life of patients with burn scars is moderate. A survey (Palmu et al., 2015) showed that the quality of life (QOL) in patients with small burns was higher than the QOL in patients with a total burn area of 30%. At the same time, most patients agree that face and hand burns have a greater impact on the patient's QOL than the actual burn area does. Salvador-Aanza et al. found that different burn patients have different changes in their body, in mental function and in other dimensions. There are many studies on the psychological function of patients after burns at home and away, but systematic research on the QOL in patients with facial burn scars is rarely reported. Some current studies have shown that the factors affecting the quality of life of patients with facial burn scars are as follows (Finnerty et al., 2016; Polychronopoulou et al., 2018): social factors [gender, marital status, occupation, and economic status (Levi et al., 2018; Spronk et al., 2018a)] disease-related factors [effects of scarring on facial function, the degree of influence, the degree of burn, and the duration of disease (Watson et al., 2018)] and psychosocial factors (stress, suppression, social support, and disability acceptance) (Garcia et al., 2016).

Disability acceptance refers to the degree to which a patient builds his or her own knowledge by integrating his or her lifestyle into an experience of dealing with disability. Patients with a higher level of disability acceptance can truly understand the meaning of existence and the ability of the group at the present stage by realizing the loss of their own value and group value due to their disabilities (Nicholls et al., 2012). Therefore, the degree of disability acceptance can predict an individual's ability to respond to attitudes against disability. The obvious exposure of facial burn scars and the importance of appearance characteristics may easily lead to a feeling of inferiority in patients with facial burn scars. Additional research efforts should be made toward understanding the relevant psychological changes after discharge from the hospital, such as the acceptance of disability.

Researchers have studied the correlation between quality of life and disability acceptance. Some studies have shown that quality of life is affected by the acceptance of disability. The level of patient disability acceptance increases with the duration of disability, and the patient is better able to adapt to life after the illness, so the quality of life also has a significant upward trend. The quality of life also increases significantly (Garcia et al., 2016). The reasons may be as follows. (a) Patients are more able to adapt to life after a longer duration of disability. (b) The effect of rehabilitation therapy is more obvious over time, and the degree of patient disability also improves. There are also studies (Nicholls et al., 2012; Baldwin et al., 2018) that indicate that there is a positive correlation between quality of life and disability acceptance.

In summary, it may have a special relationship between Quality of life and disability acceptance in patients with facial burn. However, there is no quantitative study between the two factors. Therefore, we conducted a case investigation to understand the status of the quality of life and level of disability acceptance in patients with facial burn scars and to explore the correlation between quality of life and disability acceptance and how to improve nursing care for these patients.

## Methods

### Participants

Patients with facial burn scars were investigated in an outpatient clinic of tertiary hospitals from September 2015 to February 2016. All participants are voluntary and signed informed consent before investigation.

The inclusion criteria were as follows:

1. Patient age ≥ 18 years old
2. Patients with facial damage caused only by heat, current, chemicals, laser exposure, radiation, etc., and the wound had a hypertrophic scar or keloid of >2 cm^2^ after healing
3. Conscious patients
4. Patients with an educational level of primary school and above

The exclusion criteria were as follows:

1. Patients with disabilities in other parts of the body
2. Patients with heart failure, severe liver disease, stroke, and other serious physical illnesses
3. Patients with a history of mental illness

All participants are voluntary and signed informed consent before investigation.

### Investigation

In this study, a cross-sectional survey was conducted to investigate the demographic information, quality of life, disability acceptance, and related factors of patients with facial burn scars after discharge. The questionnaires used included demographic data and investigations using the burn scars table, Burn-Specific Health Scale-Brief (BSHS-B), and acceptance disability scale (ADS). This study was approved by the ethics committee of the central hospital of Panyu District, Guangzhou.

### Measures

#### Demographic Data and Survey on Burn Scarring

An investigation was conducted by a self-designed questionnaire, which includes 10 questions (gender, age, educational level, pre-burn occupation, current occupation, marital status, place of residence, per capita monthly income of the family, average monthly treatment cost, and mode of payment for medical expenses). The disease and treatment-related information questionnaire included eight questions [cause of burn, time of scar formation, scar site, scar area, whether the patient thinks the burn scar affects facial function (e.g., facial function, sweat gland function, etc.), observation of scar by the patient, length of hospitalization, and presence of burns on other body parts].

#### Burn-Specific Health Scale-Brief (BSHS-B)

The Burn-Specific Health Scale-Brief was used to investigate the quality of life. The scale includes 9 dimensions and 40 items, including body image, work, heat sensitivity, treatment regimens, simple abilities, interpersonal relationships, hand function, affect, and sexuality. Each item of the scale has 5 rating options for each dimension score, and the Likert 5-point scale was adopted. The scale of 1–5 points for items 1–9 represents 5 levels of “failure to achieve.” The scale of 1–5 for items 10–40 represent 5 grades of “conformity,” from complete conformity to non-conformity. The lower the score of each dimension is, the lower the quality of life (Chin et al., 2018). Previous studies have shown that the scale has good reliability and validity [83], and the Chinese version of the simplified burn health scale BSHS-B has a total Cronbach's α reliability coefficient of 0.968 and Cronbach's α coefficient of 0.795~0.940 after being evaluated by relevant professionals (Gandolfi et al., 2018).

#### Acceptance Disability Scale (ADS)

The scale includes four dimensions called transformation, enlargement, containment and subordination, with a total of 50 items, in which 35 items are scored in a negative direction (one point representing “agree very much” and six points representing “disagree very much”). The remaining 15 items were scored positively. The total score of the scale ranged from 50 to 300. Low acceptance level is defined as a total score of 50–133, and scores ranging from 134 to 217 and 218 to 300 were for moderate and high acceptance levels, respectively. The subordination dimension ranges from 5 to 30 points, in which the ranges of 5–12, 13–22, and 23–30 are defined as low, moderate and high acceptance levels, respectively. The containment dimension ranges from 16 to 96 points, in which the ranges of 16–42, 43–79, and 80–96 are defined as low, moderate and high acceptance levels, respectively. The transformation dimension ranges from 15 to 90 points, in which the ranges of 15–40, 41–65, and 66–90 are defined as low, moderate and high acceptance levels, respectively. The transformation dimension ranges from 14 to 84 points, in which the ranges of 14–37, 38–61, and 62–84 are defined as low, moderate and high acceptance levels, respectively. The Cronbach's α value of this scale is 0.95 (Nicholls et al., 2012).

### Sample Size Calculation

The sample size was calculated according to the total number of scale dimensions used. The empirical formula is sample size = [Max (dimension degree) × (10~20)] × [1 + (10%~15%)]. Among the questionnaires used in this survey, the Chinese version of the BSHS-B has the highest dimensionality coefficient, with a dimensionality of 9; therefore, the dimensionality of this scale is used as the benchmark for the sample size. Considering some invalid questionnaires, the sample size required for this survey is finally defined as 130 patients.

### Quality Control

Before the investigation, the specialist nurses were given unified training on the scoring methods of the BSHS-B, ADS and disability acceptance scale, and the contents of the questionnaires were explained in the same words without guidance. Researchers and trained specialist nurses handed out and recycled all questionnaires used at the site. In the process of completing the questionnaires, unclear questions were explained, checked and supplemented in time. During the investigation, the subjects were strictly selected according to the inclusion criteria and exclusion criteria. The content and purpose of the survey were explained to the volunteers first, and then the questionnaires were collected on the premise of their informed consent. The researcher answered the questions one by one within the specified time. The investigators were required to read the answers one by one for those who could not fill in the answers by themselves, and the volunteers made their own choices without intervention.

The questionnaires were evaluated after collection. Invalid questionnaires were removed, and two teams input the data to a computer-independent order to avoid entry error. Ten percent of the data were checked through random inspection, and the unqualified rate of random inspection was controlled below 0.5%. The qualified rate of this sampling inspection was 100%.

### Data Analysis

General Demographic and Disease-Related Conditions data about Facial Burn Scar Patients were described by frequency and percentage. The Quality of Life Score was summarized as maximum, minimum, mean, and standard deviation. Each dimension of Acceptance Disability was defined as low, moderate and high acceptance and described by frequency and percentage. Differences between participants in terms of demographic characteristics, quality of life, and disability acceptance were assessed using variance analysis. Spearman correlation analysis was conducted on the quality of life score and disability acceptance.

(1) $$\begin{array}{r} \rho_{Q\text{ol},ADS}=\frac{Cov\left(Q\text{ol},ADS\right)}{\sqrt{D\left(Qol\right)}\sqrt{D\left(ADS\right)}} \end{array}$$

In, which, ρ_*Q*ol, *ADS*_, *Cov*(*Qol, ADS*), $\sqrt{D\left(Qol\right)}$, $\sqrt{D\left(ADS\right)}$ stands for the correlation, covariance between the quality of life score and disability acceptance, and their own standard variance, respectively. *P* < 0.05 was considered as significantly difference, and, all the analysis was performed using R version 3.4.3.

## Results

### General Demographic Data of Facial Burn Scar Patients

A total of 130 people were investigated in this survey, 121 valid questionnaires were recovered, and the effective questionnaire recovery rate was 93.08%. The age of the facial burn scar patients ranged from 18 to 83 years, with an average age of 42.77 ± 13.82 years old. The majority of patients were male (63.6%) and married (86%). The ratio of males to females was ~1.75:1. The education levels of patients were 12.4, 35.5, 47.9, and 4.1% for primary school, junior high school, senior high school, junior college, and undergraduate or above, respectively. In total, 82.6% of patients live in cities. Unemployed persons before burning accounted for 1.7% of the total number, while the proportion increased to 17.4% after burning. Approximately 63.6% of families have a monthly income of 2,000~4,000 yuan per capita. The average monthly treatment cost was 620.74 yuan. A total of 66.9% of patients did have medical insurance (Table 1).

**Table 1:** General demographic of facial burn scar patients (*n* = 121).

**Variable name**	**Frequency**	**Percentage (%)**
**Gender**		
Male	77	63.6
Female	44	36.4
**Age (years)**		
18–30	28	23.1
31–44	43	35.5
45–59	32	26.4
60–83	18	14.9
**Education level**		
Primary school	15	12.4
Junior high school	43	35.5
High school	58	47.9
College, undergraduate or above	5	4.1
**Pre-burn occupation**		
Workers	40	33.1
Farmers	23	19.0
Individuals, businessmen, enterprises, government	40	33.1
Housewives	11	9.1
Unemployed	2	1.7
Students	5	4.1
**Current occupation**		
Workers	28	23.1
Farmers	22	18.2
Individual, business, enterprise, government, service	32	26.4
Housewives	12	9.9
Unemployed	21	17.4
Students	5	4.1
Other	1	0.8
**Marital status**		
Unmarried	15	12.4
Married	104	86.0
Divorced/separated	2	1.7
**Residence**		
City	100	82.6
Countryside	21	17.4
**Per capita monthly income (yuan)**		
<2,000	6	5.0
2,000–4,000	77	63.6
>4,000	38	31.4
**Payment method of medical expenses**		
At their own expense	81	66.9
Medical insurance	40	33.1

### Disease Related Information of Facial Burn Scar Patients

In total, 113 people (93.4%) suffered from thermal burns. The average scar formation time of facial burns was 116.72 days, ranging from 15 to 427 days, of which 96 patients exhibited scars within 6 months and 25 exhibited scars after 6 months. Submandibular scars were the most common scar formation sites among the facial burns, accounting for 71.9% of 87 patients. Fifty-seven patients had a burn scar area ≥5 cm^2^, accounting for 47.1% of patients. A total of 7.4% of the patients believed that the impact of their scars was significant. A total of 34.7% of patients often have sensation of their facial burn scars, while only 3.3% of patients have no sensation of facial burn scars. The first hospital stay of facial burn scar patients was 2–74 days in duration, with an average of 20.31 ± 17.82 days. Three (1.7%) patients suffered from facial burns alone; 71 (58.7%) were complicated with trunk burns; 67 (55.4%) were complicated with upper limb burns; and 31 patients (25.6%) were complicated with lower limb burns (Table 2).

**Table 2:** Disease-related conditions of facial burn scar patients (*n* = 121).

**Variable**	**Frequency**	**Percentage (%)**
**Cause of bum**		
Thermal burns	113	93.4
Chemical bums	6	5.0
Others (radiation bums)	2	1.7
**Combined with burns on other body parts**		
Trunk	71	58.7
Limb	67	55.4
Lower limb	31	25.6
No combined burns	3	2.5
**Scar formation time**		
Within 6 months	96	79.3
More than 6 months	25	20.7
**Facial scar formation site**		
Frontal compartment	11	9.1
Face	26	21.5
Submandibular area	87	71.9
Eyelid	18	14.9
Mouth	10	8.3
Nasal area	1	0.8
**Scar area of facial burns**		
2~4 cm^2^	64	52.9
5~15 cm^2^	46	38.0
>15 cm2	11	9.1
**Number of days of first hospitalization**		
>9 days	36	29.8
More than 9 days	61	50.4
≥30 days	24	19.8
**Observation of facial scar by patients**		
Regular observation	42	34.7
Sometimes observation	36	29.8
Occasional observation	43	35.5
**Does the patient think the bum scar will affect his or her facial function**		
Basically no	72	59.5
Little influence	40	33.1
Great influence	9	7.4

### Quality of Life

Among 121 patients, 28.93% (35/121) had a score of quality of life greater than 145, 47.22% (57/121) had a quality of life between 130–145, and 23.97% (29/121) had a quality of life below 130. The total score of quality of life in facial burn scar patients was 137.06 ± 17.05. The scores of body image, work, heat sensitivity, simple abilities, interpersonal relationships, hand function, affect, and sexuality were 12.31 ± 2.52, 12.60 ± 3.27, 16.01 ± 3.57, 16.31 ± 2.90, 10.61 ± 2.77, 14.29 ± 1.97, 18.00 ± 4.42, 25.49 ± 4.32, and 11.44 ± 1.50, respectively (Table 3). There's significantly difference between each dimension of quality of life (*F* = 271.53, *P* < 0.01).

**Table 3:** The quality of life score of facial burn scar patients (*n* = 121).

**Item**	***X ± s***	**Average score of the single entry**
Total score	137 06 ± 17.05	3.43
Body image	12.31 ± 2.52	3 08
Work	12.60 ± 3.27	3.15
Heat sensitivity	1601 ± 357	3.2
Treatment regimens	16.31 ± 2.90	3.26
Simple abilities	10.61 ± 2.77	3.54
Interpersonal relationships	14.29 ± 1.97	3.57
Hand function	18 00 ± 4.42	3.6
Affect	25.49 ± 4.32	3.64
Sexuality	11.44 ± 1.50	3.81

### Acceptance Disability Scale (ADS)

The total score of disability acceptance was 185.68 ± 23.74. Among the scoring items, the scores for transformation, enlargement, dimension, containment and subordination were 58.64 ± 9.31, 54.12 ± 7.54, 58.04 ± 8.62, and 14.88 ± 2.75, respectively. The degree of disability acceptance and its dimensions are divided into three levels: low, medium, and high. In the distribution of the total disability acceptance score of the study subjects, 91.7% of patients were at the moderate acceptance level, while 22.3% of patients in the compliance dimension scored at the low acceptance level (Table 4).

**Table 4:** Acceptance disability level of facial burn scar patients (*n* = 121).

**Variable quantity**	**Low acceptance**		**Moderate acceptance**		**High acceptance**	
	***N***	**Percentage (%)**	***N***	**Percentage (%)**	***N***	**Percentage (%)**
Transformation	9	7.4	87	71.9	25	20.7
Enlargement	4	3.3	94	77.7	23	19
Containment	7	5.8	114	94.2	0	0
Subordination	27	22.3	91	75.2	3	2.5
Total score of disability acceptance	4	3.3	111	91.7	6	5.0

### Correlation Analysis of Quality of Life and Handicap Acceptance

Spearman correlation analysis was conducted on the quality of life score and disability acceptance. The results are shown in Table 5. The results showed that the overall quality of life score of facial burn scar patients was positively correlated with disability acceptance (*r* = 0.245, *p* = 0.007).

**Table 5:** Correlation between quality of life and disability acceptance in patients with facial burn scar (*n* = 121).

**BSHS-B dimensions**	**Transformation**	**Enlargement**	**Containment**	**Subordination**	**Total score**
Total score of quality of life	0.203^*^	0.277^**^	0.235^**^	−0.264^**^	0.245^**^
Simple abilities	0.062	0.132	0.059	−0.280^**^	0.073
Hand function	0.137	0.248^**^	0.078	−0.331^**^	0.130
Affect	0.301^**^	0.347^**^	0.357^**^	−0.287^**^	0.345^**^
Interpersonal relationships	0.160	0.165	0.184^*^	−0.299^**^	0.165
Sexuality	0.276^**^	0.402^**^	0.250^**^	−0.431^**^	0.279^**^
Body image	−0.053	0.008	0.051	0.022	0.017
Heat sensitivity	0.138	0.105	0.185^*^	−0.046	0.170
Treatment regimens	0.155	0.177	0.198^*^	−0.053	0.190^*^
Work	0.075	0.301	0.083	0.079	0.619

* *P <0.05*,

** *P <0.01*.

## Discussion

In this survey, the overall quality of life of patients with facial burn scars was divided into 77~160 points with an average score of 137.0 ± 17.05 points, indicating that the quality of life of patients with facial burn scars is at a moderate or low level. However, some researchers scored 182.43 ± 48.6 points in the study on the quality of life of patients in the burn rehabilitation period. This result may be because the face is a special area where once burned, it can easily be observed by other people, so burns on the face have a substantial effect the quality of life of patients. At the same time, facial burn scarring can increase the psychological pressure of the patient and cause great interference to his or her work and life, which may lead to a moderate-to-low quality of life (Spronk et al., 2018b).

According to the Appraisal Standard for Disability Degree of Industrial Injury and Occupational Disease of Workers, the subjects of this study have disabilities ranging from grade 4 to grade 10. The results of this study show that the total score of disability acceptance of facial burn scar patients is 185.68 ± 23.74, which is close to the score of other subjects (181.46 ± 39.45) and higher than score range of the low level of acceptance. This finding indicates that the disability acceptance of burn scar patients is at a medium level and still needs to be improved. According to the grading distribution of the total score of disability acceptance for facial burn scar patients, 91.7% of the patients were at a moderate acceptance level. The low acceptance level was 3.3%. Patients with a high acceptance level only accounted for 5%.

To our knowledge, there's still no report about the relationship between quality of life and the acceptance of disability in facial burn patients, and the acceptance of disability plays a significant role in mediating the correlation between general self-efficacy and depression/general quality of life in mild traumatic brain injury patients (Yehene et al., 2019). The results of this survey show that disability acceptance in facial burn patients is a factor affecting the quality of life of patients, and there is a positive correlation between the two factors. Similar to the research results, the reasons may be as follows. (1) The overall quality of life improvement level in patients is not only affected by the treatment level during hospitalization but also has a great correlation with the attitude in coping with their own disability. Through reasonable cognition, patients can adopt logical thinking to overcome the belittling of self-esteem, create a good life, adapt to their environments with a reasonable outlook on life, and improve their effective adaptability to their disabilities. Patients need reasonable cognition to guide adaptive behavior in the process of social reintegration after burn. Patients can identify new role orientations and self-definitions and then adopt adaptive behaviors to promote the recovery of body functions and the improvement of various skills and abilities. The improvement of disability acceptance level is conducive to further improving the physical condition of patients. (2) Improvement in the degree of disability acceptance changes patients' cognition to a certain extent, improves patients' control over their own emotions, and allows them to perceive less negative psychological emotions, thus guiding patients to actively change their self-value and attitude toward life. The continuous improvement in disability acceptance indicates that patients can actively change their self-value recognition and self-cognition, thus improving their quality of life.

Our study found that the affect dimension in the quality of life of facial burn scar patients has a correlation with all dimensions of disability acceptance, of which the correlation coefficient with the containment dimension was the largest, suggesting that the affect dimension has the closest relationship with the control dimension. According to Maslow's hierarchy of needs theory, after human beings have satisfied their physiological and safety needs, they will pursue the satisfaction of the needs of emotion and belonging (Kowal-Vern and Criswell, 2005); emotional needs are more delicate than physiological needs are, and at the same time, emotional needs have a certain relationship with individual physiological characteristics, social education, personal experience, and religious beliefs. If patients can rationally view facial burn scars and control the negative effects caused by facial burn scars so that they do not exceed the actual damage range to the body, the patient's emotion can be expressed more smoothly, and the demand level of emotion and belonging can be realized, thus improving the patient's acceptance of his or her disability. The sex life dimension of the quality of life of patients with facial burn scars is correlated with all dimensions of disability acceptance, of which the correlation coefficient with the compliance dimension is the largest, indicating that the sex life dimension is most closely related to the compliance dimension. By analyzing the reasons, patients cannot accept their current appearance changes and do not obey their current physical conditions. They still attach great importance to the facial appearance changes caused by sudden accidents and show higher attention to their own abilities and appearance. Patients will be more inclined to think that burn scars lead to the disability of their bodily functions, thus affecting their sex life (Capek et al., 2018).

## Conclusion and Clinical Significance

The quality of life of facial burn scar patients will improve with the improvement of disability acceptance level. Therefore, medical staff can improve the quality of life of patients by improving their disability acceptance level. Medical staff can assist patients to find control and management methods of the body, expand the scope of patients' values, establish a correct evaluation of their appearance, obey the changes brought about by facial burn scars, assist patients to reconstruct their internal aesthetics, and help patients to rediscover their own value orientation and meaning of life by guiding patients to formal medical institutions for scar treatment consultation and follow-up.

## Data Availability Statement

The datasets generated for this study are available on request to the corresponding author.

## Author Contributions

XZ, YL, and AH contributed to the conception and design of the study. XZ and XD organized the database. YL, CD, and YP performed the statistical analysis. XZ wrote the first draft of the manuscript. YL, XD, and CD wrote sections of the manuscript. All authors contributed to manuscript revision, read, and approved the submitted version.

### Conflict of Interest

The authors declare that the research was conducted in the absence of any commercial or financial relationships that could be construed as a potential conflict of interest.
